# Toward Predicting Motion Sickness Using Virtual Reality and a Moving Platform Assessing Brain, Muscles, and Heart Signals

**DOI:** 10.3389/fbioe.2021.635661

**Published:** 2021-04-01

**Authors:** Marco Recenti, Carlo Ricciardi, Romain Aubonnet, Ilaria Picone, Deborah Jacob, Halldór Á. R. Svansson, Sólveig Agnarsdóttir, Gunnar H. Karlsson, Valdís Baeringsdóttir, Hannes Petersen, Paolo Gargiulo

**Affiliations:** ^1^Institute of Biomedical and Neural Engineering, Reykjavik University, Reykjavík, Iceland; ^2^Department of Advanced Biomedical Sciences, University Hospital of Naples “Federico II”, Naples, Italy; ^3^Department of Anatomy, University of Iceland, Reykjavík, Iceland; ^4^Akureyri Hospital, Akureyri, Iceland; ^5^Department of Science, Landspitali University Hospital, Reykjavík, Iceland

**Keywords:** motion sickness, postural control, sea sickness, virtual reality, machine learning, heart rate, electroencephalogram – EEG, electromyography – EMG

## Abstract

Motion sickness (MS) and postural control (PC) conditions are common complaints among those who passively travel. Many theories explaining a probable cause for MS have been proposed but the most prominent is the sensory conflict theory, stating that a mismatch between vestibular and visual signals causes MS. Few measurements have been made to understand and quantify the interplay between muscle activation, brain activity, and heart behavior during this condition. We introduce here a novel multimetric system called BioVRSea based on virtual reality (VR), a mechanical platform and several biomedical sensors to study the physiology associated with MS and seasickness. This study reports the results from 28 individuals: the subjects stand on the platform wearing VR goggles, a 64-channel EEG dry-electrode cap, two EMG sensors on the gastrocnemius muscles, and a sensor on the chest that captures the heart rate (HR). The virtual environment shows a boat surrounded by waves whose frequency and amplitude are synchronized with the platform movement. Three measurement protocols are performed by each subject, after each of which they answer the Motion Sickness Susceptibility Questionnaire. Nineteen parameters are extracted from the biomedical sensors (5 from EEG, 12 from EMG and, 2 from HR) and 13 from the questionnaire. Eight binary indexes are computed to quantify the symptoms combining all of them in the Motion Sickness Index (I_*MS*_). These parameters create the MS database composed of 83 measurements. All indexes undergo univariate statistical analysis, with EMG parameters being most significant, in contrast to EEG parameters. Machine learning (ML) gives good results in the classification of the binary indexes, finding random forest to be the best algorithm (accuracy of 74.7 for I_*MS*_). The feature importance analysis showed that muscle parameters are the most relevant, and for EEG analysis, beta wave results were the most important. The present work serves as the first step in identifying the key physiological factors that differentiate those who suffer from MS from those who do not using the novel BioVRSea system. Coupled with ML, BioVRSea is of value in the evaluation of PC disruptions, which are among the most disturbing and costly health conditions affecting humans.

## Introduction

Postural control (PC) is a central nervous system (CNS) feedback control system that governs human upright stance and gives a platform for locomotion and task-driven behavior, as well as several autonomic responses. The PC system works on a subconscious level and is based on continuous CNS input from visual, vestibular, proprioceptive, and somatosensory receptors Massion (1994). The CNS then processes this information to direct (efferent signals) both somatic (muscular) and autonomic (blood pressure etc.) responses. The PC system can be disturbed in two ways: the first one is a disease disruption (lost function) at all levels, and the second is a physiological “overstimulation” (increased function), which gives rise to motion sickness (MS).

### State of the Art

Motion sickness is experienced by those who passively travel and is more common in women and at a young age. Although there are great individual differences, sex and age are both predictors of MS and motion sickness susceptibility (MSS) in general populations, probably due to gene–environment interaction Golding (2006a).

In addition, MS and MSS also fluctuate across age, i.e., in general, humans from 2 years of age begin to feel motion sick during traveling, peaking at 13 years of age and declining postpubertal (Bos et al., 2007; Huppert et al., 2019).

One of the best-known manifestations of MS is seasickness Petersen (2012). Due to modern technology, humans have faced new MS situations such as spaceflights [space sickness Crampton (1990)] or when playing computer games, including the phenomenon of “cybersickness” in virtual reality (VR) environments LaViola (2000). MS is a polysymptomatic disorder, where the primary symptoms are nausea and vomiting, but sweating, facial pallor, increased salivation, drowsiness, and dizziness are also frequent Golding (2006a). There is varying susceptibility among the general population, but all those with a fully or partially functional vestibular system can experience

MS. Females report higher incidence in MS history (higher frequency and severity of symptoms) and are more susceptible to seasickness, simulator sickness, and visually induced MS than males of the same age (Flanagan et al., 2005; Lawther and Griffin, 1988; Turner, 1999).

Two main theories regarding the pathogenesis of MS exist. The “sensory conflict theory” (SCT) Reason (1978) states that MS is caused by conflict between visual, vestibular, and/or somatosensory inputs. In the case of passive travel, such as being a passenger in a car or on a ship, the physical motion perceived by the vestibular system does not match the expected signals from the visual system. Sensory conflict can also occur due to a purely visual stimulus, as can be experienced by people during VR simulations who may perceive a visual movement, but vestibular signals do not match this. Recent studies report possible “sensory conflict neurons” in the brainstem and cerebellum (Oman and Cullen, 2014; Cohen et al., 2019) and also brain networks that mediate nausea and vomiting Yates et al. (2014b), which appear to further support the sensory conflict theory. A second theory of pathogenesis in MS is the “postural control theory” Riccio and Stoffregen (1991). It states that prolonged postural instability precedes the subjective symptoms of MS, i.e., that MS is directly brought on by an inability to control the posture during motion rather than a detection of any sensory conflict. The ability to remain bipedal/upright is crucial to human survival and MS appears to be closely linked to postural instability; studies have shown that greater postural instability or increased body sway correlates with greater MSS (Cobb, 1999; Tal et al., 2010).

Regardless of the underlying pathophysiology, CNS adaptive signals as well as efferent signals involved in the corrective processes, preceding and during MS, are measurable via various methods. Some studies have looked into possible correlations between MS levels and physiological biosignals such as electroencephalography (EEG), electrogastrography (EGG), electro-oculography (EOG), skin conductivity, heart rate (HR), blood pressure, body temperature, and cerebral blood oxygen demand visualized in functional magnetic resonance imaging. Relationships between the levels of MS experienced by subjects have been demonstrated in various EEG, EGG, and eye movement studies. Koohestani et al. (2019) give an overview of objective biosignal measures in MS research. Objective kinematic measures such as center of pressure (COP) are also documented as having relationships to MS levels in the literature (Thurrell and Bronstein, 2002; Weech et al., 2018) as well as spectral characteristics of spontaneous sway, which have been measured as a possible objective measurement for a predictive MS parameter Laboissière et al. (2015).

Objective measures can be useful for tracking the onset of MS, as it may be possible to use biosignals to predict the likelihood of the subject experiencing MS. Therefore, a subject’s MSS can be linked to measurable physiological or kinematic parameters in some cases by correlating the objective measurements with a standardized subjective MSS test/experienced MS level test. This is crucial for all further genetic studies on MS.

Motion sickness susceptibility is generally assessed by means of a questionnaire: subjective reporting of experienced levels of typical MS symptoms during biosignal measurement is a method used extensively in recent experimental studies (Kennedy et al., 1993; Golding, 2016; Mazloumi Gavgani et al., 2018). Correlation of various biosignals and subjective reporting of MS levels is a task to which machine learning (ML) is actively contributing. EEG has been used as a technique to correlate biosignal measurements with MS levels in multiple subjects using ML for VR-related MS Li et al. (2020). Ko et al. (2011) used neural network ML algorithms to estimate patient’s MS level based on the EEG power spectra from possible stimulated brain areas. Li et al. (2019) also studied EEG, COP, and head and waist motion markers correlated to a subjective MS questionnaire using ML following visually induced MS. Wang et al. (2019) used postural difference measures pre- and post-visually induced MS calculated with a deep long short term memory model. These studies used visually induced MS exclusively for estimating physiological response in virtual environments. Hell and Argyriou (2018) also used ML to predict MS using a VR rollercoaster simulation tool and a neural network architecture predicting MS and the intensity of roller coasters in order to improve the gaming experience.

### Scientific Goal and Proposed Experiment

**FIGURE 1:**
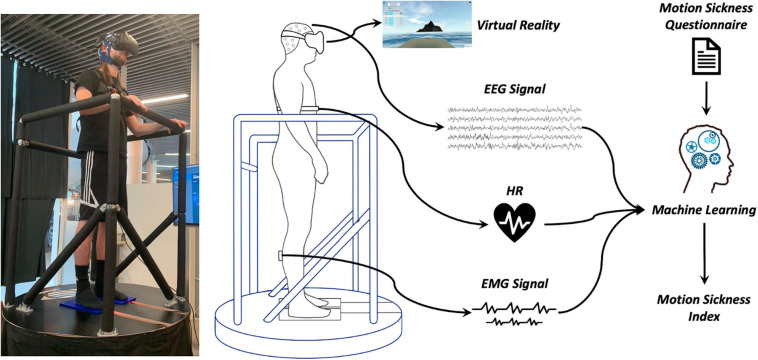
BioVRSea structure: the moving platform, shown in a photo with a subject on the left, is combined with a rough sea VR scenario and with EEG, EMG, and HR bio-signal acquisition.

In this paper, we present the results from the first study using a new seasickness measurement platform called BioVRSea. This system is a sailing simulator that records, in synchronized fashion, heart, muscle, and brain signals (Figure 1). The participants wear the VR goggles showing a rough sea scenario. The movement of the ship on the waves in the VR scenario is coupled to the moving platform and the frequency and amplitude of the VR wave motion is synchronized with the platform motion. Subjective and objective MS levels are assessed by a questionnaire while biosensors measure EEG, electromyography (EMG), and HR of the subject. The creation of a database allows the implementation of various statistical and ML algorithms with the aim of correlating the biometric results with new indexes that combine the various symptoms of MS, having as main novelty the EEG application and interpretation in association with VR and moving platform inducing MS, linked to other biosignals.

## Materials and Methods

The biosensors used in this study are 64-channel EEG, 2-channel EMG, and HR chest monitor.

This first study is based on data acquired from 28 subjects (age: 23.8 ± 1.2), 22 women and 6 men (ethic approval by the Icelandic Bioethics Commission – Number: VSN-20-101 – May 2020). Each participant is measured three times (except one subject who underwent only two protocols) using different protocols based on the amplitude and frequency of the simulated waves. From each protocol, we extract 19 parameters associated with HR, EEG, and EMG signals. Moreover, after every protocol, the subject answers the Motion Sickness Susceptibility Questionnaire (MSSQ) Golding (2006b) based on the self-evaluation of 13 different neurophysiological conditions. A total of 83 datasets [(28 × 3) - 1] constitute the final database of our study.

### BioVRSea Measurement’s Protocols

The VR software (Virtualis, VR, France) dynamically visualizes a virtual environment as if the subject is out on the open sea on a little boat. A moving platform (Virtualis VR, France) mimics the waves according to the simulated environment (Figure 1). The operator can set frequency (between 0.5 and 3 Hz) and amplitude of the waves (from 0 to 2). During the simulation, we vary the amplitude of the platform movements from 0% up to 100%. The platform allows fast (tailored) movements in 0°−45°−90°−135°−180°−225°−270°−315°−360° (linear acc. not available) coupled to synchronized visual VR movements.

**FIGURE 2:**
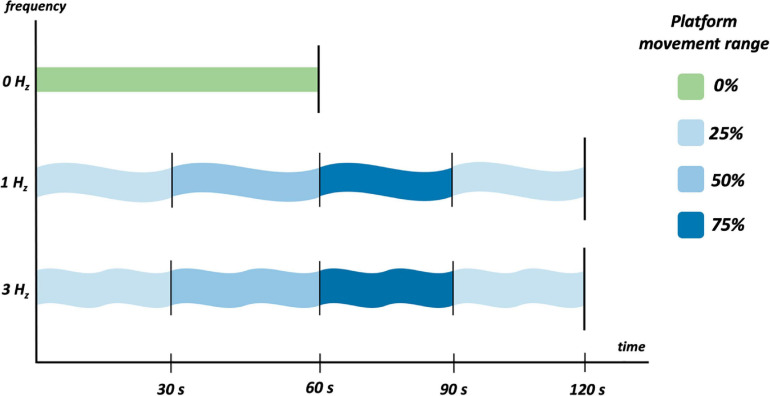
The three acquisition protocols that each patient has been subjected to.

Three different protocols are implemented in this study (Figure 2):

• 0 Hz, null wave amplitude. Sea simulation is not performed during this protocol. The subject remains in an upright position on the platform for 60 s, observing mountains surrounded by lights in a dark environment through the goggles.
• 1 Hz, and wave amplitude = 0.6. This sea simulation protocol is divided into four parts, 30 s each with different platform movement amplitudes: 25, 50, 75, and again 25%. Total time: 120 s.
• 3 Hz, and wave amplitude = 0.5. This sea simulation protocol is divided into four parts, 30 s each with different platform movement amplitudes: 25, 50, 75, and again 25%. Total time: 120 s.

The first protocol mentioned (0 Hz) is the non-movable (platform stable) pre-test (baseline) sampling where the subjects can relax. This is done before the other two protocols (1 and 3 Hz) where the subjects during the movements have to grab onto the protection bars that they have in front. The eyes must be opened during all the three protocols.

The selection of these frequencies was based on two main reasons. The first reason is to only act upon one of MS etiologic theory: multiple theories have been listed to explain MS, and the SCT is easily the leading perspective. Frequencies below 1 Hz are not considered because they might act upon the additional Postural Instability Theory, which is rooted in perception of lower <0.5 Hz frequencies Riccio and Stoffregen (1991). The second reason is to ensure that an easy scenario (1 Hz) is available to reduce the risk of falling, as well as a harder one (3 Hz) to ensure sufficient movement to trigger MS.

### Data Acquisition

During each protocol, heart, muscle, and brain data are acquired using the following technologies:

• HR is measured using a heart chest sensor (Polar Electro, Kempele, Finland, sampling frequency of 1,000 Hz).
• Muscle electrical activities from the lower limbs are acquired using two wireless EMG sensors (sampling frequency of 1,600 Hz) placed on the gastrocnemius of each leg (Kiso ehf, Reykjavik, Iceland).
• Brain electrical activity is measured using a 64-channel dry electrode cap (sampling frequency of 500 Hz) from AntNeuro, Hengelo, Netherlands.

### Feature Extraction

Electromyography data processing was performed using Matlab_2020b (MathWorks Inc., Natick, Massachusetts, United States). The EMG signal was filtered with a 50th-order FIR bandpass filter with cutoff frequencies at 40 and 500 Hz. A fast Fourier transform was then used to obtain the frequency spectrum. The relative power spectral density (PSD) was then calculated for five frequency bands, equally distributed from 40 to 500 Hz, resulting in five parameters per leg. Finally, the integral of the rectified EMG signal for each leg was calculated and divided by the sample size, resulting in one parameter per leg. A total of 12 EMG-related features is thus computed.

The EEG was recorded using a 64-dry electrode channel system with an EOG electrode placed below the right eye and a ground electrode placed on the left side of the neck. Data pre-processing and analysis were performed with Brainstorm Tadel et al. (2011) and Matlab_2020b.

The data were re-referenced using the common average reference. A high-pass and a low-pass filter were set, respectively, from 0.1 and 40 Hz. Bad channels were manually removed when EEG voltage was higher than 300 μV. If more than 20% of the channels showed too much noise or incorrect signal, the whole trial was rejected. The signals were digitized in segments of 30 s, within 1-Hz and 3-Hz protocols. DC offset correction was performed, and baseline correction was applied using the 0-Hz segment. Channels marked as bad were removed and interpolated. Individual trials were visually inspected and rejected when indicative of excessive muscle activity, eye movements, or other artifacts.

The PSD was computed for each epoch with Welch’s method, with the following frequency bands: delta (0.5–4 Hz), theta (4–8 Hz), alpha (8–13 Hz), beta (13–35 Hz), and low gamma (LG) (35–40 Hz). The relative power of each band was then computed and averaged across all channels, obtaining a total of five EEG-related features.

Finally, from the HR signal, we calculate two features: HR average and standard deviation.

This results in a total of 19 biometric features for each acquisition protocol (Table 1).

**TABLE 1:** Description of the 19 biometric parameters that compose the database.

**Biometric parameter**	**Description**
EEG – Delta	Relative power spectra between frequency band 0.5–4 Hz
EEG – Theta	Relative power spectra between frequency band 4–8 Hz
EEG – Alpha	Relative power spectra between frequency band 8–13 Hz
EEG – Beta	Relative power spectra between frequency band 13–35 Hz
EEG – LG	Relative power spectra between frequency band 35–40 Hz
EMG – L area	Integral of the rectified EMG signal of left gastrocnemius divided by the sample size
EMG – R area	Integral of the rectified EMG signal of right gastrocnemius divided by the sample size
EMG – L 40-132	Left gastrocnemius relative PSD in the 40–132 Hz frequency band
EMG – L 132-224	Left gastrocnemius relative PSD in the 132–224 Hz frequency band
EMG – L 224-316	Left gastrocnemius relative PSD in the 224–316 Hz frequency band
EMG – L 316-408	Left gastrocnemius relative PSD in the 316–408 Hz frequency band
EMG – L 408-500	Left gastrocnemius relative PSD in the 408–500 Hz frequency band
EMG – R 40-132	Right gastrocnemius relative PSD in the 40–132 Hz frequency band
EMG – R 132-224	Right gastrocnemius relative PSD in the 132–224 Hz frequency band
EMG – R 224-316	Right gastrocnemius relative PSD in the 224–316 Hz frequency band
EMG – R 316-408	Right gastrocnemius relative PSD in the 316–408 Hz frequency band
EMG – R 408-500	Right gastrocnemius relative PSD in the 408–500 Hz frequency band
HR average	Heart rate average
HR std	Heart rate standard deviation

Table 2 shows the objective physiological measurement differences for all the subjects between the first static protocol and the other two, the light one (1 Hz) in green and the hard one (3 Hz) in red. The arrows show how the values of the single EEG, EMG, and HR data rise or fall during the protocols. For example, it is possible to notice how the EMG values for both legs at low frequencies increase for the most patients, while they decrease at high frequencies. On the opposite, the EEG values do not follow such a regular trend.

**TABLE 2:**
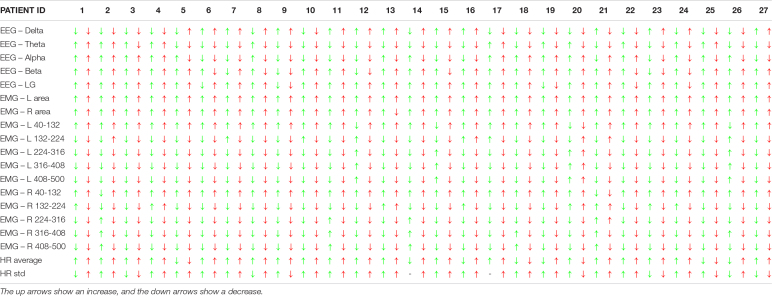
Difference of the objective brain, muscle, and health bio measurements between the first static protocol and the light (1 Hz – green) and the hard one (3 Hz – red) for all the patients (the one that did not perform the 3-Hz protocol is not included).

### Motion Sickness Questionnaire and Relative Indexes

At the end of every protocol the subjects were asked to fill out a questionnaire regarding their MS symptoms. The questionnaire is based on the MSSQ proposed by Golding (2006b). The subjects must give a score between zero and two for 13 typical MS symptoms: general discomfort, dizziness and vertigo, stomach awareness, sweating, nausea, salivation, burping, headache, fullness of head, blurred vision, fatigue, eye strain, and difficulty focusing.

We define a total of eight binary indexes considering the MSSQ answers.

General discomfort (I_*G*__*en*__*D*__*is*_, 1st) and Dizziness and Vertigo (I_*D*__*izz*_, 2nd) are considered as independent and individual indexes. Stomach awareness, sweating, nausea, salivation, and burping are considered together as stomach-related to create the Stomach-related Index (I_*Sto*__*m*_, 3rd). Headache, fullness of head, and blurred vision together produce the Head Index (I_*Head*_, 4th) while fatigue, eye strain, and difficulty focusing contribute to the Fatigue Index (I_*Fatig*_, 5th). I_*Stom*_, I_*Fatig*_, and I_*Head*_ are computed as binary indexes following these steps: first, we compute the average from the individual responses of each index; second, we calculate the maximum among the averages; and third, we divide the cohort into two groups (below and above 1/3 of the maximum). For I_*GenDis*_ and I_*Dizz*_, we apply only steps 2 and 3 using the direct response instead of the average.

Moreover, we established two more indexes, Physiological/Vegetative Index (I_*PV*,_ 6th) and Neurological/Muscle Strain Index (I_*NM*,_ 7th). I_*PV*_ is based on the previously outlined steps from the responses from sweating, salivation, nausea, burping, stomach awareness, and general discomfort conditions. Similarly, the I_*NM*_ is based on fatigue, eye strain, difficulty focusing, headache, fullness of head, blurred vision, and again general discomfort conditions.

The last index is called Motion Sickness Index (I_*MS*_, 8th), and it is based on the weighted sum SumMS of all the MSSQ answers (Eq. 1) and steps 2 and 3.

SumMS=(0,2⋅GenDisc+0,2⋅Dizz&Vert+0,2⋅

∑(StomAwe,Nausea,Sweat,Saliv,Burp)+0,2⋅

∑(Fatigue,EyeStr,DiffFocus)+0,2⋅

(1) ∑(Headache,FullHead,BlurrVis))

In Table 3, it is possible to see the percentage of MSSQ answers and indexes for the entire cohort. It is possible to identify general discomfort, sweating, nausea, and vertigo as the most significant indexes with over 20% of responses being 2, which is the highest possible value. Salivation and burping, conversely, are the least significant with a percentage lower than 5% providing a response of value 2.

**TABLE 3:** Percentage of the MSSQ answers for each symptom, and percentage of zeros and ones for the eight computed indexes.

**MSSQ Symptoms**	**0 (%)**	**1 (%)**	**2 (%)**	**Index**	**0 (%)**	**1 (%)**	**Index**	**0 (%)**	**1 (%)**	**Index**	**0 (%)**	**1 (%)**
**Gen. Discomfort**	43.4	30.1	26.5	**I_*GenDis*_**	43.4	56.6						
**Sweating**	43.4	32.5	24.1									
**Salivation**	79.5	15.7	4.8				**I_*PV*_**	63.9	36.1			
**Nausea**	61.4	14.5	24.1	**I_*Stom*_**	62.6	37.4						
**Burping**	94.0	6.0	0.0									
**Stomach Awern.**	66.3	18.1	15.7									
**Fatigue**	67.5	16.3	13.2							**I_*MS*_**	61.4	38.6
**Eye Strain**	53.8	26.9	19.2	**I_*fatig*_**	56.6	43.4						
**Diff. Focusing**	62.7	22.9	14.5									
**Headache**	55.4	25.3	19.3				I_NM_*	59.0	41.0			
**Full. Of Head**	63.9	22.9	13.2	**I_*Head*_**	57.8	42.2						
**Blurr. Vision**	61.4	26.5	12.0									
**Dizziness - Vertigo**	54.2	24.1	21.7	**I_*Dizz*_**	54.2	45.8						

*I_*NM*_ includes also General Discomfort.

Table 4, similarly to Table 2, shows the increase or decrease of the value of the subjective given answer to the questionnaire using the colored arrows. It is possible to see that some patients, like numbers 10, 11, 14, and 22, have an increase of the symptom for both the 1-Hz and the 3-Hz protocols while others do not show any significant difference. Subject number 16 shows an increase only with the 3-Hz protocol, confirming the strong influence of the wave frequency on the body.

**TABLE 4:**
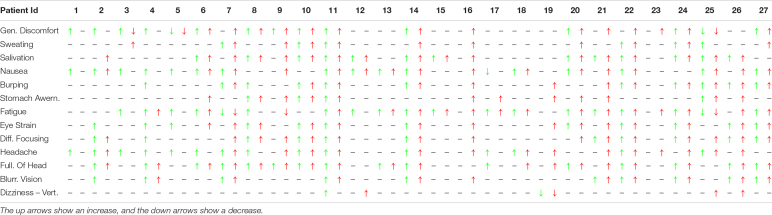
Difference of the subjective MS symptoms between the first static protocol and the light (1 Hz – green) and the hard (3 Hz – red) for all the patients (the one that did not perform the 3-Hz protocol is not included).

### Statistical Analysis

All the parameters extracted from EEG, EMG, and HR underwent a non-parametric statistical univariate explorative analysis in order to understand whether there was a statistically different grouping by I_*GenDis*_, I_*Dizz*_, I_*Stom*_, I_*Head*_, I_*Fatig*_, I_*PV*_, I_*NM*_, and I_*Ms*_. All the indexes underwent univariate statistical analysis through the Mann–Whitney test.

### ML Tool and Algorithms

The ML analysis was performed by using KNIME Analytics Platform (v. 4.2.0), which is a well-known platform in the field of biomedical studies, as it is considered the best choice for advanced users of ML Tougui et al. (2020) Several studies have been performed in clinical settings: for radiomics studies in oncology (Ricciardi et al., 2019; Romeo et al., 2020), for fetal monitoring (Improta et al., 2019; Ricciardi et al., 2020b), for investigating some relationships in ophthalmology (D’Addio et al., 2019; Improta et al., 2020), and in cardiology Ricciardi et al. (2020a).

The following algorithms were implemented through the platform: Random Forests (RF), Gradient Boosting tree (GB), Ada-Boosting of decision tree (ADA-B), Support Vector Machine (SVM), K Nearest Neighbor (KNN), and Multilayer Perceptron (MLP). The first three are based on a structure made up of nodes (starting point of the tree, which indicates an attribute), leaves (the question to be answered, which is the final label), and branches (connecting the nodes) and showed good results in different studies (Recenti et al., 2020; Ricciardi et al., 2020c). There are many criteria for splitting up the records: the gain ratio was used in this study Safavian and Landgrebe (1991). RF and GB are two empowered versions of the decision tree; they apply the ensemble learning methods of randomization, bagging, and boosting to make the weak learner stronger (Breiman, 2001; Friedman, 2001). SVM and KNN are two instance-based algorithms (Keller et al., 1985; Suykens and Vandewalle, 1999); the former assigns the class to the test data based on their distance from similar training data while the latter is capable of solving problems that have to do with overfitting, small datasets, and non-linear and/or high-dimensional data; it can also be used for both classification and regression. It aims to find the best hyperplane that divides the dataset into two classes. MLP consists of a form of neural network with an input layer, one or more hidden layers, and an output layer. The training is usually achieved by using the algorithm backpropagation of errors or some of its variants Riedmiller and Braun (1993).

The most employed evaluation metrics were used to assess the performance of the algorithms into the classifications tasks: accuracy, sensitivity, specificity, and Area Under the Curve Receiver Operating Characteristics (AUCROC) Hossin and Sulaiman (2015). All these metrics were computed using the K-Fold Cross Validation Kohavi (1995) with *k* = 10 using 10 different seeds. This means that the database is divided into 10 groups and each of them is used in turn as the test group while the other nine are used for the training of the model. Using 10 different seeds allows the creation of different 10-fold divisions, which allows a better exploration of the database and achieving the best results.

## Results

### Statistical Analysis Results

Table 5 shows the results of the statistical tests that assess the significance of the 19 parameters with the eight binary MSSQ indexes. Interestingly, only 4 out of 19 parameters never show a significance.

**TABLE 5:** Significance of the 19 biometric parameters calculated with the univariate statistical analysis (Mann–Whitney test) for all the eight indexes.

	**I_*GenDis*_**	**I_*Stom*_**	**I_*Fatig*_**	**I_*Head*_**	**I_*Dizz*_**	**I_*PV*_**	**I_*NM*_**	**I_*MS*_**
**EEG – Delta**	0.734	0.903	0.790	0.383	0.841	0.529	0.993	0.667
**EEG – Theta**	0.934	0.880	0.388	0.613	0.400	0.927	0.184	0.181
**EEG – Alpha**	0.713	0.865	0.769	0.620	0.577	0.560	0.971	0.888
**EEG – Beta**	0.393	0.510	0.236	0.050*	0.194	0.105	0.130	0.335
**EEG – LG**	0.508	0.247	0.229	0.029*	0.070	0.087	0.081	0.217
**EMG – L area**	0.114	0.001***	0.004**	0.001***	0.001***	0.001***	0.001***	0.001***
**EMG – R area**	0.157	0.004**	0.001***	0.001***	0.001***	0.004**	0.001***	0.001***
**EMG – L 40–132**	0.274	0.029*	0.006**	0.001***	0.001***	0.040*	0.004**	0.011**
**EMG – L 132–224**	0.941	0.492	0.274	0.079	0.165	0.444	0.285	0.449
**EMG – L 224–316**	0.247	0.031*	0.006**	0.001***	0.001***	0.090	0.010**	0.013**
**EMG – L 316–408**	0.236	0.025*	0.012**	0.003**	0.001***	0.043*	0.003**	0.011**
**EMG – L 408–500**	0.286	0.023*	0.029*	0.003**	0.001***	0.032*	0.002**	0.009**
**EMG – R 40–132**	0.040*	0.006**	0.003**	0.001***	0.001***	0.002**	0.001***	0.008**
**EMG – R 132–224**	0.044*	0.027*	0.051*	0.035*	0.015**	0.005**	0.008**	0.100
**EMG – R 224–316**	0.079	0.026*	0.012**	0.007**	0.002**	0.037*	0.007**	0.040*
**EMG – R 316–408**	0.139	0.030*	0.006**	0.001***	0.001***	0.039*	0.003**	0.012**
**EMG – R 408–500**	0.118	0.020*	0.003**	0.001***	0.001***	0.018*	0.001***	0.003**
**HR average**	0.001***	0.219	0.082	0.314	0.012**	0.037*	0.010**	0.042*
**HR std**	0.040*	0.451	0.213	0.149	0.251	0.264	0.249	0.136

*Significant at 0.05. **Significant at 0.01. ***Significant at 0.001.

The EEG Beta and LG showed significance only for the individuals suffering from headache, fullness of head, and blurred vision (I_*Head*_), while no other significances were found for an EEG parameter.

The amplitude/area of EMG on both sides achieved a significance for all the conditions except for General Discomfort (I_*GenDis*_). Similarly, excluding a few cases, the power spectrum of the EMG obtained a significance for almost all the conditions except in the band of 132–224 for the left side, which is never significant.

The HR Average was significant according to all indexes excluding I_*Stom*_, I_*Fatig*_, and I_*Head*_ while HR std showed statistical significance only according to I_*GenDis*_.

The I_*GenDis*_ index was the index that showed the least number of significances for the analyzed parameters; only EMG – R 40–132 and 132–4 Hz and HR parameters achieved significant results according to this index. On the other hand, I_*Head*_, I_*Dizz*_, and I_*NM*_ were the indexes according to which the biometric parameters show the greatest number of significant results (respectively, 13 and 12).

Finally, 11 out of 19 parameters show a significant result according to the overall I_*MS*_: 10 EMG-related features, 1 HR-related feature, and no EEG feature.

### ML Results

The ML analysis focuses on the binary classification of physiological, neurological, and general MS conditions based on the MSSQ responses. We performed the classification of the following index previously defined:

(1) The Physiological/Vegetative Index (I_*PV*_),
(2) The Neurological/Muscle Strain Index (I_*NM*_),
(3) The MS Index (I_*MS*_).

We assessed these conditions using six different algorithms, finding RF to yield the best results (Table 6).

**TABLE 6:** Evaluation metrics after the classification ML analysis for I_*PV*_, I_*NM*_, and I_*MS*_.

**Index**	**Algorithms**	**Accuracy**	**Sensitivity**	**Specificity**	**AUCROC**
I_*PV*_	RF	**75.9**	77.5	74.4	**0.815**
	GB	74.7	75.0	74.4	0.705
	ADA-B	**75.9**	85.0	67.4	**0.781**
	SVM	60.2	62.5	58.1	0.603
	KNN	57.8	60.0	55.8	0.573
	MLP	49.4	77.5	23.3	0.642
I_*NM*_	RF	**79.5**	74.3	83.3	**0.832**
	GB	69.9	71.4	68.8	0.711
	ADA-B	73.5	80.0	68.8	0.746
	SVM	60.2	51.4	66.7	0.590
	KNN	67.5	65.7	68.8	0.694
	MLP	45.8	80.0	20.8	0.737
I_*MS*_	RF	**74.7**	59.4	84.3	**0.801**
	GB	72.3	68.8	74.5	**0.803**
	ADA-B	71.1	71.9	70.6	**0.765**
	SVM	55.4	43.8	62.7	0.532
	KNN	67.5	59.4	72.5	0.670
	MLP	53.0	40.6	60.8	0.681

As regards I_*PV*_ and I_*NM*_, the RF was the best algorithm for classifying both indexes; an accuracy of 75.9% with an AUCROC of 0.815 was achieved for I_*PV*_ while an accuracy of 79.5% with an AUCROC of 0.832 was obtained. The highest sensitivity (85.0%) was obtained by the ADA-B for the physiological index, while the highest sensitivity was achieved by RF (74.4%). As regards the neurological index, the best sensitivity (80.0%) was achieved by the ADA-B while the best specificity (83.3%) was obtained by RF. The MLP was the worst algorithm to perform both the classifications since it reached the lowest accuracy (respectively, 49.4 and 45.8%) while the lowest AUCROC was reached by KNN for the physiological index (0.573) and by SVM for the neurological index (0.590).

**FIGURE 3:**
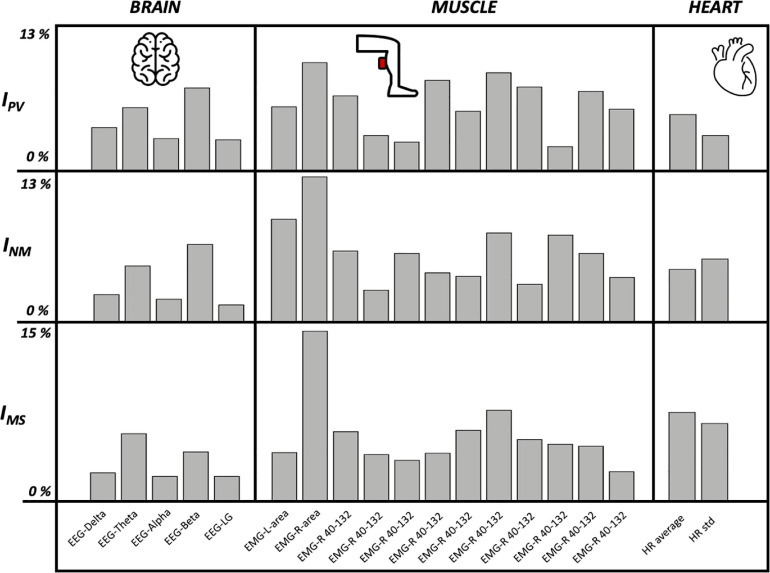
Brain, muscle, and heart feature importance for I_*PV*_, I_*NM*_, and I_*MS*_ using Random Forest algorithm.

The feature importance analysis (Figure 3) shows that parameters extracted from EMG were the most important ones by far for the classification of both indexes. The first EEG-based features can be found in the 5th place in the ranking while the first HR-based features can be found after the 10th place. Moreover, it has to be highlighted that the top three features in Figure 3 for these indexes are all statistically significant also in the previous univariate analysis (Table 5).

Concerning I_*MS*_, the overall model for the indexes is good enough considering accuracies greater than 70.0%, AUCROC greater than 0.800, and the number of trials (equal to 83) that does not allow us to analyze a large dataset; indeed, a greater number of subjects would allow the improvement of the evaluation metrics of the models.

The feature importance analysis (Figure 3 and Table 7) highlighted novel results for I_*MS*_: the seasickness can be strongly linked to features extracted from EMG (the top two were area and frequency analysis in the range 40–132) and HR-based (average and standard deviation were at the third and fourth place). On the other hand, another important and surprising result is the low importance of all the features extracted from the EEG, they were below the seventh place in the final ranking (this also for the other indexes except EEG-Beta which is quite relevant for I_*PV*_ and I_*NM*_). This can be explained by the fact that a dry cap EEG was used for the acquisition. More noise was detected and led to a lower signal quality. Channels had to be rejected and could not be interpolated, leading to an averaged PSD on less channels. This can be one of the reasons of the low significance related to EEG features.

**TABLE 7:** Feature importance (%) for IPV, INM, and IMS using Random Forest algorithm.

	**I_*FV*_**	**I_*NM*_**	**I_*MS*_**
EEG – Delta	3.74	2.37	2.47
EEG – Theta	5.46	4.84	5.87
EEG – Alpha	2.78	1.97	2.16
EEG – Beta	7.18	6.71	4.29
EEG – LG	2.68	1.48	2.16
EMG – L area	5.56	8.88	4.23
EMG – R area	**9.39**	12.54	**14.77**
EMG – L 40–132	6.51	6.12	6.04
EMG – L 132–224	3.07	2.76	4.06
EMG – L 224–316	2.49	5.92	3.56
EMG – L 316–408	**7.85**	4.24	4.18
EMG – L 408–500	5.17	3.95	6.16
EMG – R 40–132	**8.52**	7.7	**7.9**
EMG – R 132–224	7.28	3.26	5.36
EMG – R 224–316	2.11	7.5	4.96
EMG – R 316–408	6.9	5.92	4.78
EMG – R 408–500	5.36	3.85	2.57
HR average	4.89	4.54	**7.72**
HR std	3.07	5.43	6.76

## Discussion

Postural control is central in governing upright posture in humans. PC failure is dual, firstly pathological disruption leading to clinical difficulties where symptoms of vertigo, dizziness, imbalance, and falling are prominent Dakin and Bolton (2018). Secondly, an overstimulation of the PC system may precipitate a series of symptoms of discomfort known as MS Golding (2016). As in PC diseases, there are many objective measurements to be used in the diagnosis of these diseases. On the other hand, there are limited ways to objectively measure MS. Questionnaires are used to evaluate the incidence of subjective symptoms associated with MS, most often nausea, pallor, vomiting, sweating, headache, lightheadedness, and body discomfort Balk et al. (2013). There is an urgent need for objective measurement to evaluate MS, as it threatens human well-being when one is situated in a motion-rich environment. It is also critical to objectively distinguish people into MS-prone and non-MS-prone individuals. This is possible using questionnaires Golding (2006b), but having an objective way to discriminate these two groups is of great value when comes to genetic research.

In this study, we use the BioVRSea research setup and focus on EEG, EMG, and HR bio-signals associated with subjective MS symptoms. Our EEG-coupled results show significant difference in brain neural networking in individuals indicating subjective symptoms of headache, fullness of the head, and blurred vision (I_*Head*_). In an earlier study, we showed that open eyes trials reflect a greater number of significant differences in EEG absolute spectral power across all bands during both adaptation and habituation. This suggests that following both acute and prolonged proprioceptive perturbation, cortical activity may be up-regulated with the availability of visual feedback Barollo et al. (2020). These results generally support our prior hypothesis that the visual recognition of instability may play a critical role in governing cortical processes requisite for PC Edmunds et al. (2019). These results underline the importance of visual information in PC and simultaneously open up the VR afferent link in PC perturbations. Being able to couple these subjective symptoms, i.e., headache, fullness of the head, and blurred vision, to objective intracranial activity is crucial in clinical context and opens up ways for VR-coupled biosignal evaluation of PC pathologies Maire et al. (2017). This is in keeping with many other studies performed on motion and CNS triggers of head-related symptoms. Jang et al. (2020) identified that the alpha band was linked to VR sickness, with a decrease of the absolute power during the experiment, followed by an increase during the recovery, highlighting a negative correlation with the MSSQ score. Kim et al. (2005) detected that in the case of cybersickness, the severity of the symptoms was positively correlated with the delta wave, and negatively with the beta waves. It is interesting to see that, in our study, despite the low significance of EEG regarding the different indexes chosen, the power associated to the beta band is the parameter presenting the most importance in I_*PV*_ and I_*NM.*_ This corroborates the fact that beta band is related to MS symptoms and is a feature that should be investigated in MS studies. Our results do not enable to drawing of hypotheses regarding the other power bands.

Our observations have indicated that definite vection does not necessarily result in visually induced MS (you can have very compelling vection but no visually induced MS), but at the same time, most participants who get sick also report vection. Our intentions are to verify the relationship between MS and visually induced MS, although there are some participants that get sick on the platform but never experience MS in the real world. They probably have not experienced enough rough waters and therefore we do not expect false positive/negative.

To be able to evaluate the relationship between BioVRSea biosignals and subjective MS symptoms, the use of ML was necessary. This study clearly shows the benefit of ML; indeed, it allowed us to achieve two aims: first, the possibility to model several biometric parameters extracted from three types of signals (EEG, EMG, and HR) in order to be able to classify/distinguish patients suffering from seasickness according to these features; second, the feature importance analysis allowed us to further confirm the statistical results by ranking the features according to their contribution to the classification task. Moreover, as regards the ML models, the RF was the most reliable among all the implemented ones (Table 6).

The amplitude of the EMG signals in both legs showed significant difference regarding all conditions except general discomfort (I_*GenDis*_). That indicates that almost all subjective symptoms of MS showed correlation with changes in EMG. This is in context with the fact that all human efforts initiated to prevent falls, i.e., acute or long-term vertigo and dizziness, are mediated through postural stabilizing muscles Perrin et al. (2018). Some studies used EMG measurements to analyze the behavior related to MS, with sensors placed on the abdominal muscles Shafeie et al. (2013) and EMG combined with cervical vestibular myogenic potential to study the effect of scopolamine for the seasickness treatment Tal et al. (2016). As far as we know, no study found significance related to EMG in the lower limbs to quantify MS. This is an important piece of information regarding our BioVRSea research setup and is a promising single tool to objectively extract MS sufferers. On the other hand, this is not surprising as the prime effector in PC is aimed at muscles maintaining the upright posture and simultaneously avoiding falls. Our BioVRSea research setup might answer several clinical questions related to strategies used to prevent falls in patients with PC pathologies.

The HR parameters were significantly associated with the symptom of General Discomfort (I_*GenDis*_). The General Discomfort symptom is general in its nature and does not specifically point to MS. On the other hand, the triggered MS discomfort relates to an escalated sense of generalized panic in severe MS conditions, which is well capable of creating extreme cardiovascular deviations Yates et al. (2014a). This is expected as HR is probably the best-known biosignal associated with numerous physical as well as pathological conditions, particularly of a PC nature.

These results can be used to have quite a whole vision of the body reaction to induced MS. This total vision can be used to help the pathological patients and the people that are more prone to MS planning an eventual rehabilitative therapy. Future ideas are to use more physiological measurements like blood oxygenation, skin sweating, and force used on the legs for the equilibrium. All these actual and future body parameters coupled with the BioVRSea system and ML are of value in further evaluation of PC disruptions, which are probably the most disturbing and costly health conditions affecting humans.

### Limitations

Of course, the study has some limitations. The first is the small number of subjects that limits also the possibility of obtaining higher evaluation metrics in the ML analysis. The second is the type of population that has been analyzed in this research because it was limited regarding age and health status; all the subjects were young and healthy. Further studies could increase the number of subjects, which would allow improvements in the performance of ML and include in the population more diverse subjects. We use dry electrodes for the EEG acquisition resulting in high noise signal, which we believe has limited the value of the associated EEG parameters in both statistical significance and ML. The use of a wet cap EEG for further acquisitions is a potential improvement.

Of course, the VR view that the investigated individual visualizes standing on a virtual small vessel is not a true scenario of working environment at sea but is nevertheless capable of creating MS sensation at least in experienced sailors (verbal statements after being on platform).

## Data Availability Statement

The raw data supporting the conclusions of this article will be made available by the authors, without undue reservation.

## Ethics Statement

The studies involving human participants were reviewed and approved by the Icelandic Bioethics Committee – Number: VSN-20-101. The patients/participants provided their written informed consent to participate in this study.

## Author Contributions

MR, CR, RA, HP, and PG wrote the manuscript. MR and CR conceived the acquisition protocols and conducted the acquisitions with HS, SA, and GK. MR and CR conceived and computed the eight indexes with the support of PG. RA and GK performed the EEG analysis. HS, SA, and VB performed the EMG analysis. CR performed the machine learning analysis. IP performed the univariate statistical analysis. MR designed the tables and the figures and formatted the manuscript; Figure 1 is created by VB and MR. DJ reviewed and made additions to the manuscript. HP contributed to the medical knowledge. HP and PG conceived the original idea of quantifying motion sickness using bionsensors and virtual reality. PG coordinated the work. All authors contributed to the article and approved the submitted version.

## Conflict of Interest

The authors declare that the research was conducted in the absence of any commercial or financial relationships that could be construed as a potential conflict of interest.

## Abbreviations

ADA-B: ada-boosting
AUCROC: area under the curve receiver operating characteristics
CNS: central nervous system
EEG: electroencephalography
EGG: electrogastrography
EMG: electromyography
EOG: electro-oculography
GB: gradient boosting tree
HR: heart rate
I_*Dizz*_: dizziness and vertigo index
I_*Fatig*_: fatigue index
I_*GenDis*_: general discomfort index
I_*Head*_: head index
I_*MS*_: motion sickness index
I_*NM*_: neurological/muscle strain index
I_*PV*_: physiological/vegetative index
I_*Stom*_: stomach-related index
KNN: K nearest neighbor
LG: low gamma
ML: machine learning
MLP: multilayer perceptron
MS: motion sickness
MSS: motion sickness susceptibility
MSSQ: motion sickness susceptibility questionnaire
PC: postural control
PSD: power spectral density
RF: random forests
SCT: sensory conflict theory
SVM: support vector machine
VR: virtual reality.
